# No-Reference Image Quality Assessment with Multi-Scale Orderless Pooling of Deep Features

**DOI:** 10.3390/jimaging7070112

**Published:** 2021-07-10

**Authors:** Domonkos Varga

**Affiliations:** Independent Researcher, H-1139 Budapest, Hungary; varga.domonkos7@upcmail.hu

**Keywords:** no-reference image quality assessment, deep learning, convolutional neural networks

## Abstract

The goal of no-reference image quality assessment (NR-IQA) is to evaluate their perceptual quality of digital images without using the distortion-free, pristine counterparts. NR-IQA is an important part of multimedia signal processing since digital images can undergo a wide variety of distortions during storage, compression, and transmission. In this paper, we propose a novel architecture that extracts deep features from the input image at multiple scales to improve the effectiveness of feature extraction for NR-IQA using convolutional neural networks. Specifically, the proposed method extracts deep activations for local patches at multiple scales and maps them onto perceptual quality scores with the help of trained Gaussian process regressors. Extensive experiments demonstrate that the introduced algorithm performs favorably against the state-of-the-art methods on three large benchmark datasets with authentic distortions (LIVE In the Wild, KonIQ-10k, and SPAQ).

## 1. Introduction

Image quality assessment has crucial importance in the acquisition, processing, analysis, and reproduction of digital images. Hence, how to design an appropriate algorithm for objectively evaluating the perceptual quality of digital images is particularly important. With the advent of large image quality assessment databases [1,2], data-driven deep learning methods have become popular in this field. In this study, with the aim of providing an accurate image quality assessment scheme, we propose an innovative deep structure based on pretrained convolutional neural networks (CNN).

Objective image quality assessment algorithms can be divided into full-reference, reduced-reference, and no-reference groups depending on the availability of the reference image. Full-reference image quality assessment (FR-IQA) methods require full access to the reference image, while no-reference image quality assessment (NR-IQA) algorithms do not need the reference image. On the other hand, reduced-reference image quality assessment (RR-IQA) algorithms require partial information about the reference images.

### 1.1. Related Work

There is a large number of NR-IQA algorithms in the literature [3,4,5,6]. Moreover, many different approaches have been taken. Before the appearance of different deep learning techniques, NR-IQA research was mainly focused on the application of traditional machine learning techniques or quality-aware image feature extraction. For example Lv et al. [7] and Li et al. [8] utilized neural networks for quality prediction. Specifically, Lv et al. [7] elaborated multi-scale difference of Gaussian (DoG) features and trained a deep neural network for perceptual image quality prediction, while Li et al. [8] extracted features from the input images via Shearlet transform and image quality prediction was treated as a classification problem using neural networks. Many research papers focused on the construction of natural scene statistics (NSS) features [3,9,10,11]. The main idea behind NSS-based approaches is that the human visual system (HVS) has been evolved through natural selection, and hence it must integrate detailed information about the statistical regularities of our visual environment. Over the years, many NSS features have been introduced in the spatial and transformation domains. For example, Moorthy et al. [10] extracted NSS features in the wavelet domain over several scales and orientations. On the other hand, Saad et al. [12] extracted NSS features in the block discrete cosine transform (DCT) domain. Mittal et al. [3] proposed a feature extraction method using spatial luminance statistics. Ye and Doermann [13] utilized codebook learning to extract quality-aware features from images. Specifically, a Gabor filter was applied as local feature extractor and codebooks were complied from the extracted features. In [14,15], quality-aware feature vectors were derived from the fist digit distribution in wavelet coefficients, DCT coefficients, and singular values, the image entropy, and the image moments. Next, the compiled feature vectors were mapped onto perceptual quality scores using Gaussian process regression.

With the development of deep learning, more and more research has begun to experiment with different deep learning techniques to elaborate effective NR-IQA algorithms. For example, Kang et al. [4] worked out a NR-IQA method that estimated the perceptual quality of digital images based on image patches and a trained CNN. First, the input gray-scale image was normalized. Second, non-overlapping patches were selected from the normalized image. Subsequently, each patch was sent to the input of a particular CNN which consisted of five layers. Specifically, the last layers were regression layers which estimated the perceptual quality of the image patches. Finally, the overall quality was obtained by averaging the patches’ subscores. Similarly, Li et al. [16] trained a CNN on image patches but combined CNNs and Prewitt magnitude on a segmented image to predict image quality. Specifically, weights were determined for each image patch based on the Prewitt magnitude map. In contrast, Hou et al. [17] trained a discriminative deep model to classify NSS features into five quality categories, i.e., excellent, good, fair, poor, and bad. After classification, five grades were assigned to the input image with corresponding probabilistic confidences. Subsequently, the final quality was determined by a pooling step. In contrast, Ravela et al. [18] first identified the type of image distortion with the help of a CNN. Second, the perceived image quality degradation was predicted for each distortion type. Finally, the perceptual quality was obtained by a weighted average. Similarly, Fan et al. [19] applied a CNN first for image distortion identification. Subsequently, other CNNs were trained for each image distortion type using image patches cropped from the input images. Finally, a fusion procedure was applied to obtain the perceptual quality score of the whole input image. Other researchers applied pretrained CNNs, such as AlexNet [20] or VGG16 [21], as a feature extractor to elaborate effective quality-aware features. For instance, Bianco et al. [22] extracted feature vectors from random patches of an input image by a fine-tuned pretrained CNN model. Subsequently, the extracted feature vectors were mapped onto subscores with a trained support vector regressor. To obtain the perceptual quality, the mean of these subscores was taken. In contrast, Gao et al. [23] extracted resolution independent features from multiple layers of an AlexNet model via global minimum and maximum pooling. Similarly to the method of Bianco et al. [22], the layer-wise feature vectors were mapped onto subscores with a trained support vector regressor and the average of the subscores was taken to get the perceptual quality. In [24], deep features were extracted from multiple Inception modules of pretrained CNNs, concatenated together, and mapped onto quality scores.

First, Lin and Wang [25] applied generative adversarial networks [26] (GAN) for NR-IQA. Specifically, the task of the generative network was to generate a hallucinated reference (distortion free) image for the distorted, input image. Subsequently, the information extracted from the hallucinated, reference image was paired with those extracted from the distorted image to predict the perceptual image quality. Similarly, Ma et al. [27] proposed a GAN for NR-IQA. In contrast to other methods, the GAN was applied to predict the primary content of a distorted image and based on this, a multi-stream quality network was trained to quantify the effects of content, distortion, and degradation dependencies.

### 1.2. Contributions

Image representation has been in the focus of the image processing and computer vision community [28]. Advances in deep learning have motivated the application of deep features extracted from convolutional neural networks to image quality assessment [29] and other image processing tasks [30,31,32]. Inspired by the idea of spatial pyramid pooling [33], a deep architecture is introduced in this study where deep features are extracted from the input image at multiple scales to improve the effectiveness of feature extraction. Unlike other deep architectures [18,22,23], a multi-scale orderless pooling of deep features is elaborated where feature extraction is performed beginning from local random image patches at multiple scales. Unlike our previous method [24], the focus is on constructing an architecture that extracts deep features from multiple scales of an image rather than examining the effects of deep features extracted from multiple layers of a deep CNN. Extensive experiments have been carried on three large benchmark IQA databases (LIVE In the Wild [34], KonIQ-10k [1], and SPAQ [2]) to demonstrate that the proposed method is able to outperform the state-of-the-art.

### 1.3. Structure

The rest of the paper is organized as follows. Section 2 gives a detailed description of the proposed method. Section 3 describes the employed publicly available benchmark databases used in this study, defines the evaluation criteria, demonstrates experimental results and analysis, and introduces a comparison to other state-of-the-art algorithms. Finally, the conclusions are drawn in Section 4.

## 2. Proposed Method

**Figure 1:**
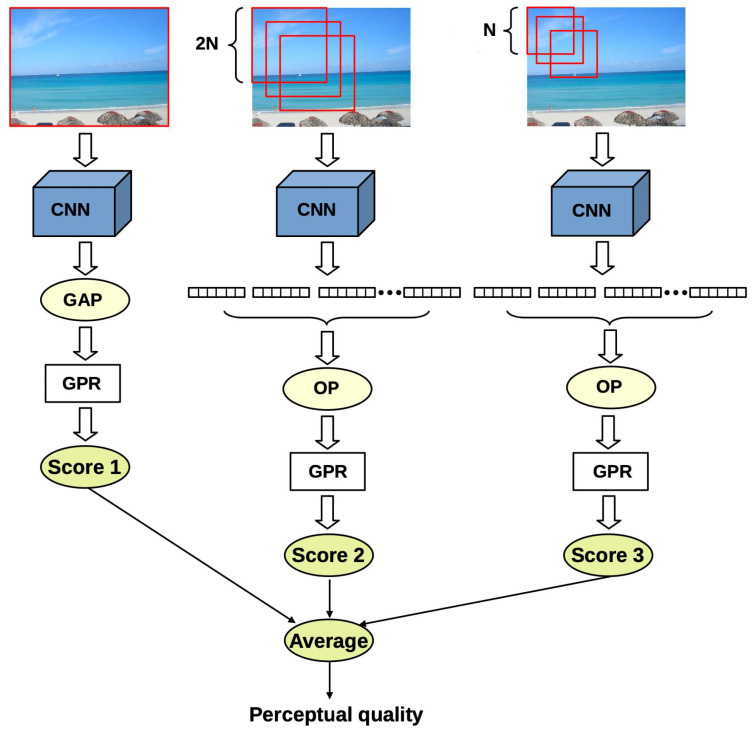
Block diagram of the proposed method. The proposed method extracts deep features from the input image at three different scales. The first scale corresponds to the whole image. At the second scale, square random patches are extracted whose size is the double that of the applied pretrained CNN’s input size, while the patches’ size corresponds to the input of the pretrained CNN at the third scale.

Inspired by the idea of spatial pyramid pooling [33], a deep architecture is proposed which extracts feature vectors from multiple image patches at multiple scales starting from the whole image. The feature vectors of the individual scales are pooled together and mapped onto perceptual quality scores independently from each other through Gaussian process regression (GPR). The general overview is depicted in Figure 1. The proposed architecture has three different scale levels, corresponding to the original size of the input image, the input size of the applied pretrained CNN, and to the double input size of the CNN, respectively. To extract the deep features from the different scales of the input image, we made experiments with three different CNN networks pretrained on the ImageNet [35] database in a parameter study (Section 3.3).

**Figure 2:**
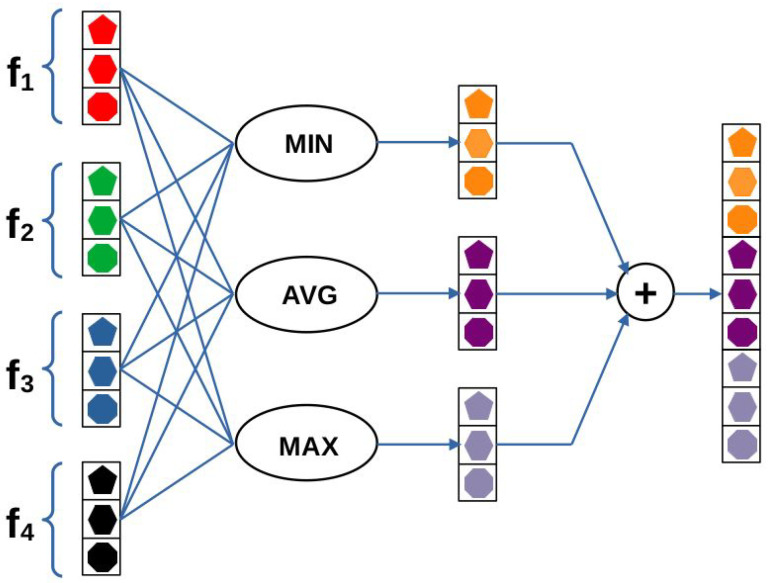
Illustration for orderless pooling of feature vectors. The core components of this structure correspond to a set of statistical functions, i.e., minimum, average, and maximum. Each function is applied to the set of input feature vectors and the outputs of the functions are concatenated. The figure is best viewed in color.

Given an input image for the first level, we simply extract the feature maps from a given layer of a pretrained CNN. To compile feature vectors, the extracted feature maps are run through global average pooling (GAP) layers. GAP layers are applied to decrease the spatial dimensions of the feature maps into single values by simply taking the average of all values within a feature map. This way, feature vectors can be created for the first level whose dimensions are independent from the input image’s size and only depend on the applied pretrained CNN architecture. Since GAP layers perform a very extreme type of pooling, important information for IQA may disappear in the case of high-resolution images. That is why two more scales were added to the network. In the second scale, square random patches are extracted from the input image whose sizes are twice as much as the input size of the applied feature extractor pretrained CNN. As in the first scale, feature maps are extracted from each image patch via the pretrained CNN, and feature vectors are complied by running the deep feature maps through GAP layers. To compile one feature vector that characterizes the whole scale, orderless pooling is introduced in this study (depicted in Figure 2). Let us suppose that we have $N_{f}$ feature vectors with length *M*. Let $f_{i}^{\left(j\right)}$ stand for the *i*th entry of the *j*th image patch’s feature vector. In the proposed orderless pooling method, minimum, average, and maximum operators are defined as follows:

(1) $$F_{i}^{min}=\min_{j=1,\ldots ,N_{f}}f_{i}^{\left(j\right)},\;\;\;\;\;i=1,\ldots ,M,$$

(2) $$F_{i}^{avg}=\frac{1}{N_{f}}\sum_{j=1,\ldots ,N_{f}}f_{i}^{\left(j\right)},\;\;\;\;\;i=1,\ldots ,M,$$

(3) $$F_{i}^{max}=\max_{j=1,\ldots ,N_{f}}f_{i}^{\left(j\right)},\;\;\;\;\;i=1,\ldots ,M.$$

In the proposed orderless pooling method, the median operator was not applied, since we did not experience any performance improvement when the median operator was added. The reason for that is the results of the average and median operators being identical or nearly equal in most feature maps of the base CNN.

The output of the orderless pooling layer is the concatenation of the outputs of the operators defined above:

(4) $$F=F^{min}⊕F^{avg}⊕F^{max},$$

where ⊕ stands for the concatenation operator. Similar to the second scale, square random patches are sampled from the input image in the third scale. However, the size of the patches corresponds to the input size of the applied pretrained CNN. As a consequence, feature vectors can be directly extracted from the image patches through the fully-connected layers of pretrained CNNs. To compile one feature vector that characterizes the third scale, orderless pooling is applied as in the previous case. In our implementation, 15 random image patches are extracted in the second scale and 20 patches are sampled in the third scale.

The feature vectors of the three scales are mapped onto perceptual quality scores using GPRs with rational quadratic kernel functions. To obtain the perceptual quality of the entire image, the average of the three scales’ predictions is taken. GPRs are non-parametric kernel-based probabilistic models [36]. The rational quadratic kernel function allows the modeling of data at multiple scales [37]. Moreover, the rational quadratic kernel function corresponds to the infinite sum of radial basis function kernels with various characteristic length scales. The kernel is given by:

(5) $$k\left(x_{i},x_{j}\right)={\left(1+\frac{d{\left(x_{i},x_{j}\right)}^{2}}{2\alpha l^{2}}\right)}^{-\alpha },$$

where α stands for the scale mixture parameter, *l* corresponds to the length scale of the kernel, and d(·,·) denotes the Euclidean distance function.

## 3. Experimental Results and Analysis

In this section, our experimental results and analysis are presented. First, we describe the applied benchmark datasets used in this study in Section 3.1. Second, the definitions of the applied performance indices and implementation details are given in Section 3.2. Subsequently, we analyze the experimental results of our proposed method with parameters’ design and compare it with other state-of-the-art methods in Section 3.3 and Section 3.4, respectively. In Section 3.5, a cross database is presented where the generalization ability of the examined NR-IQA algorithms are tested. Finally, the computational times of feature extraction are compared in Section 3.6.

### 3.1. Datasets

The detailed information about the publicly available image quality assessment databases used in this study are summarized in Table 1. As one can see, four large databases containing images with either authentic or artificial distortions were used in our evaluation. The KonIQ-10k [1] database consists of 10,073 digital images with authentic distortions which were evaluated in a large-scale crowdsourcing procedure with 1467 crowd workers. The images of this database were selected from the YFCC100m database [38]. Similarly, Ghadiyaram et al. [34] evaluated the perceptual quality of digital images in a crowdsourcing experiment, but the images were collected from photographers who were asked to take photos by different mobile device cameras. The SPAQ [2] database contains 11,125 various high-resolution images taken by a wide variety of mobile cameras. In contrast to KonIQ-10k [1] and LIVE In the Wild [34] (CLIVE), the captured images were assessed in a laboratory environment. In contrast to the above-mentioned databases, TID2013 [39] contains 25 reference images and 3000 distorted images which were derived from the reference images using 24 types of distortions at five different distortion levels. The images were evaluated by 971 human observers in five different countries (Finland, France, Italy, Ukraine, and the USA).

The main features of the used publicly available IQA databases are summarized in Table 1.

### 3.2. Evaluation Criteria and Environment

Pearson linear correlation coefficient (PLCC), Spearman rank order correlation coefficient (SROCC), and Kendall rank order correlation coefficient (KROCC) were used to evaluate the prediction performance of our method and other state-of-the-art algorithms. These coefficients were calculated between the ground-truth and predicted scores. A correlation coefficient of 1 corresponds to perfect prediction, while 0 correlation coefficient indicates no correlation. Specifically, the predicted scores were mapped to the subjective ratings using the following nonlinear logistic function before calculating the PLCC:

(6) $$Q=\beta_{1}\left(\frac{1}{2}-\frac{1}{e^{-\beta_{2}\left(Q_{p}-\beta_{3}\right)}}\right)+\beta_{4}Q_{p}+\beta_{5},$$

where $Q_{p}$ and *Q* stand for the predicted and mapped scores, respectively. The $\beta_{i}\left(i=1,\ldots ,5\right)$ variables are the fitting parameters.

Given paired data $\left(x_{1},y_{1}\right),\ldots ,\left(x_{m},y_{m}\right)$, PLCC is defined as:

(7) $$PLCC\left(x,y\right)=\frac{\sum_{i=1}^{m}\left(x_{i}-\bar{x}\right)\left(y_{i}-\bar{y}\right)}{\sqrt{\sum_{i=1}^{m}{\left(x_{i}-\bar{x}\right)}^{2}}\sqrt{\sum_{i=1}^{m}{\left(y_{i}-\bar{y}\right)}^{2}}}$$

where $\bar{x}=\frac{1}{m}\sum_{i=1}^{m}x_{i}$ and $\bar{y}=\frac{1}{m}\sum_{i=1}^{m}y_{i}$. On the other hand, SROCC can be defined as:

(8) $$SROCC\left(x,y\right)=PLCC\left(\mathrm{rank}(x),\mathrm{rank}(y)\right)$$

where the rank(·) operator returns with a vector whose *i*th element is the rank of the *i*th element in the input vector. The definition of KROCC between x and y is

(9) $$KROCC\left(x,y\right)=\frac{n_{c}-n_{d}}{\frac{1}{2}n\left(n-1\right)}$$

where *n* is the length of the input vectors, and $n_{c}$ and $n_{d}$ denote the number of concordant and discordant pairs between x and y, respectively.

The main features of the computer configuration used in our experiments are summarized in Table 2. The proposed method was implemented and tested in MATLAB R2020a relying on the functions of the Deep Learning Toolbox, the Image Processing Toolbox, and the Statistics and Machine Learning Toolbox.

To evaluate our proposed method and other state-of-the-art algorithms, the IQA benchmark database containing authentic distortions (CLIVE [34], KonIQ-10k [1], SPAQ [2]) were divided simply into training (appx. 80% of images) and test sets (appx. 20% of images). On the other hand, the TID2013 [39] database was divided with respect to the reference images to avoid semantic content overlap between the training (appx. 80%) and test sets (appx. 20%). In the followings, median PLCC, SROCC, and KROCC values are reported which were measured over 100 random train–test splits.

### 3.3. Parameter Study

In this subsection, a parameter study is carried out to present experimental results with respect to several different types of pretrained CNNs and layers. Although the proposed method can be generalized to any other pretrained CNNs, AlexNet [20], VGG16 [21], and VGG19 [21] were chosen as base CNNs in this study, since they are a very common choice in IQA [22,23]. A comprehensive evaluation of all possible pretrained CNNs is out of the scope of this study.

**Figure 3:**
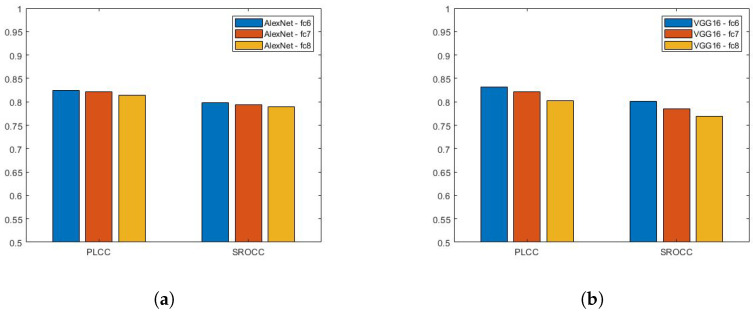

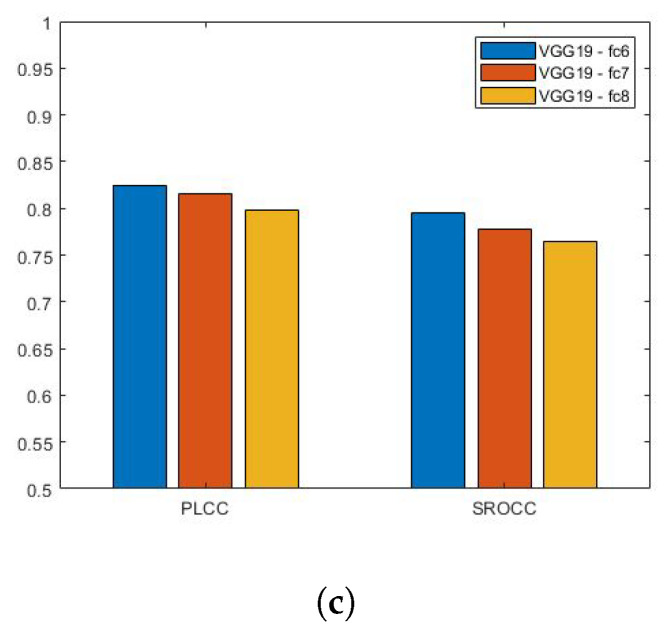
Performance comparison of deep features extracted from (**a**) AlexNet [20], (**b**) VGG16 [21], and (**c**) VGG19 [21]. Median Pearson’s linear correlation coefficient (PLCC) and Spearman’s rank order correlation coefficient (SROCC) values were measured over 100 random train–test splits.

The main characteristics of the applied pretrained CNNs are summarized in Table 3. AlexNet [20] was a breakthrough in the history of deep learning. It consists of five convolutional and three fully-connected layers. Moreover, it introduced the ReLU activation function and the dropout technique. The main novelty of VGG16 and VGG19 [21] was that the input image is passed through a stack of convolutional layers where the size of the filters is 3×3 all over. Bianco et al. [22] extracted deep features from the fc7 layer of AlexNet [20] like pretrained CNNs. In this study, we examine the features of the last three fully-connected layers of AlexNet [20], VGG16 [21], and VGG19 [21]. The results are summarized in Figure 3. It can be observed that deep features extracted from the fc6 layer of the VGG16 [21] network provide the highest correlation values in terms of PLCC and SROCC. As a consequence, the fc6 layer of VGG16 [21] was chosen as a source of deep features in the proposed architecture. Moreover, this architecture is codenamed MSDF−IQA in the following sections and subsections.

#### 3.3.1. Effect of the Number of Patches

As already mentioned in Section 2, the number of patches on the second and third scale were set to 15 and 20, respectively. In this paragraph, experimental results are presented with respect to different number of image patches on CLIVE [34] and KonIQ-10k [1]. The results are summarized in Table 4 and Table 5. First, we intuitively set the number of patches to 3 and 4 on the second and third scale, respectively. Next, the number of patches were increased by 3 and 4 in five steps, respectively. Over 15 and 20 patches, we experienced no performance gain. This is why 15 and 20 were chosen for the number of patches on the second and third scale, respectively.

#### 3.3.2. Effect of the Scales

As described in Section 2 and depicted in Figure 1, the proposed method extracts deep features from the input image at three different scales. Specifically, the first scale corresponds to the whole image, while, at the second scale, image patches are sampled whose sizes correspond to the double input size of the applied CNN. Finally, at the third scale, the size of the patches is exactly the same as the input of the CNN. In this paragraph, we present the performance results of the individual scales. The results are summarized in Table 6. It can be seen that the features of Scale 3 significantly outperform those of other scales. Moreover, considering information from all scales improves the performance of image quality prediction.

### 3.4. Comparison to the State-of-the-Art

To compare the proposed *MSDF-IQA* algorithm with other state-of-the-art methods, twelve NR-IQA algorithms (DeepRN [40], BLIINDS-II [11], BMPRI [41], BRISQUE [3], CurveletQA [42], DIIVINE [43], ENIQA [44], GRAD-LOG-CP [45], NBIQA [46], PIQE [47], OG-IQA [48], SSEQ [49]) were collected whose original source codes are available online. Moreover, we reimplemented the deep learning based BLIINDER [23] method (available at: https://github.com/Skythianos/BLIINDER (accessed on on 8 July 2021). To evaluate the proposed *MSDF-IQA* algorithm and the other state-of-the-art methods, the applied benchmark IQA databases (CLIVE [34], KonIQ-10k [1], SPAQ [2]) were divided into a training (appx. 80% of images) and a test set (appx. 20% of images). The TID2013 [39] database was divided into a training and a test set with respect to the reference images to avoid semantic content overlap between these two sets. Moreover, median PLCC, SROCC, and KROCC values are reported in this study which were measured over 100 random train–test splits.

The experimental results of our and the other state-of-the-art algorithms on authentic distortions are summarized in Table 7 and Table 8. It can be seen that the proposed *MSDF-IQA* is able to outperform the other twelve state-of-the-art algorithms on three very large IQA benchmark databases (CLIVE [34], KonIQ-10k [1], and SPAQ [2]) containing authentic distortions. Table 9 contains the results measured on TID2013 [39]. Since TID2013 contains images with small resolution (512 × 384), the implementation of MSDF-IQA was modified by considering 1.5× of the base CNN’s input size, instead of 2× on the second scale. As it can be seen, the proposed method achieves the third best result on TID2013 [39] behind BLIINDER [23] and DeepRN [40] in terms of PLCC.

To prove that the achieved results are statistically significant, one-sided t-tests were carried out between the results of *MSDF-IQA* and those of other state-of-the-art methods. The results of the significance tests are summarized in Table 10. It can be observed that the introduced method is able to produce significantly better results than the examined state-of-the-art algorithms.

**Figure 4:**
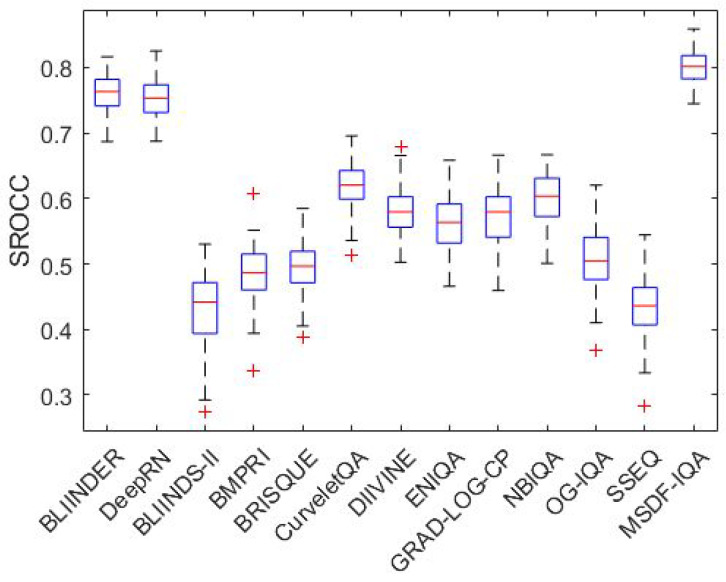
Boxplots of measured SROCC values on CLIVE [34]. The examined NR-IQA algorithms were evaluated over 100 random train–test splits.

**Figure 5:**
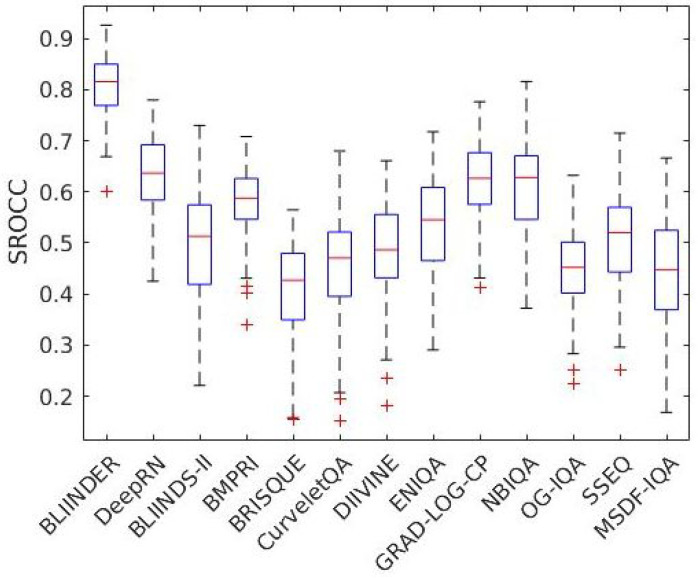
Boxplots of measured SROCC values on TID2013 [39]. The examined NR-IQA algorithms were evaluated over 100 random train–test splits with respect to the reference images.

Figure 4 and Figure 5 illustrate the boxplots of the measured SROCC values of the examined NR-IQA algorithms on CLIVE [34] and TID2013 [39] databases, respectively. Specifically, on each box, the red central mark denotes the median. Moreover, the blue bottom and top edges of the boxes denote the 25th and 75th percentiles, respectively. The most extreme values, which are not considered as outliers, are indicated by whiskers. Outliers are depicted by ’+’.

### 3.5. Cross Database Test

Resolution, spatial information, and image semantics may influence the performance of machine learning based NR-IQA algorithms. Hence, the generalization ability of NR-IQA methods are often evaluated in cross database tests, where the methods are trained on one database and tested on another one. In this study, we have KonIQ-10k [1] and CLIVE [34] IQA databases for this purpose. Namely, the examined methods were trained on KonIQ-10k [1] and tested on CLIVE [34]. The results of the cross database test are summarized in Table 11. It can be seen that the proposed is able to outperform all the other examined state-of-the-art NR-IQA methods in this test.

### 3.6. Computational Complexity of Feature Extraction

In this subsection, we compare the computational times of feature extraction using the computer configuration described in Table 2. The results for CLIVE [34], KonIQ-10k [1], SPAQ [2], and TID2013 [39] are summarized in Table 12. It can be observed that the traditional machine learning and hand-crafted feature based OG-IQA [48] and GRAD-LOG-CP [45] are the fastest methods. On the other hand, the extraction of deep features can be carried out efficiently due to GPU acceleration. This is why the examined deep learning based methods (BLIINDER [23], DeepRN [40], and *MSDF-IQA*) are able to outperform several traditional methods. Moreover, the resolution of input images has lesser impact on the computational times of feature extraction if the input image and the base CNN fit into the GPU memory.

## 4. Conclusions

In this paper, a novel architecture for NR-IQA was proposed that—inspired by the idea of spatial pyramid pooling—extracts deep features from the input image at multiple scales to improve the effectiveness of feature extraction using convolutional neural networks. Specifically, we started to extract deep activation features from local random image patches at multiple scales. The base scale was the entire image and, at finer scales, the local details of the image were captured. The extracted deep features were mapped onto perceptual quality scores with the help of trained Gaussian process regressors. Extensive experiments demonstrated that the introduced method is able to perform favorably against state-of-the-art methods on three large benchmark IQA datasets with authentic distortions, such as LIVE In the Wild [34], KonIQ-10k [1], and SPAQ [2].

To facilitate the reproducibility of the presented results, the source code of the proposed method and test environments written in MATLAB R2020a are available at: https://github.com/Skythianos/MSDF-IQA (accessed on 8 July 2021).

## Figures and Tables

**Table 1:** Publicly available IQA benchmark databases used in this paper.

Database	Year	#Distorted Images	Resolution	Environment
TID2013 [39]	2013	3000	512 × 384	laboratory
CLIVE [34]	2015	1162	500 × 500	crowdsourcing
KonIQ-10k [1]	2018	10,073	1024 × 768	crowdsourcing
SPAQ [2]	2020	11,125	∼4000 × 4000	laboratory

**Table 2:** Computer configuration applied in our experiments.

Computer model	STRIX Z270H Gaming
CPU	Intel(R) Core(TM) i7-7700K CPU 4.20 GHz (8 cores)
Memory	15 GB
GPU	Nvidia GeForce GTX 1080

**Table 3:** On the ImageNet [35] database pretrained CNNs used in this study.

Network	Depth	Size	Parameters (Millions)	Image Input Size
AlexNet [20]	8	227 MB	61.0	227 × 227
VGG16 [21]	16	515 MB	138	224 × 224
VGG19 [21]	19	535 MB	144	224 × 224

**Table 4:** Performance comparison on the effect of image patches’ number on CLIVE [34]. Median PLCC, SROCC, and KROCC values were measured over 100 random train–test splits.

CLIVE [34]				
#Patches—Scale2	#Patches—Scale3	PLCC	SROCC	KROCC
3	4	0.825	0.797	0.604
6	8	0.827	0.800	0.607
9	12	0.828	0.800	0.607
12	16	0.831	0.801	0.607
15	20	0.831	0.801	0.607

**Table 5:** Performance comparison on the effect of image patches’ number on KonIQ-10k [1]. Median PLCC, SROCC, and KROCC values were measured over 100 random train–test splits.

KonIQ-10k [1]				
#Patches—Scale2	#Patches—Scale3	PLCC	SROCC	KROCC
3	4	0.888	0.872	0.690
6	8	0.895	0.878	0.696
9	12	0.898	0.882	0.701
12	16	0.899	0.884	0.703
15	20	0.901	0.885	0.703

**Table 6:** Performance comparison of different scales on CLIVE [34]. Median PLCC, SROCC, and KROCC values were measured over 100 random train–test splits.

	CLIVE [34]			KonIQ-10k [1]		
	PLCC	SROCC	KROCC	PLCC	SROCC	KROCC
Scale 1	0.810	0.778	0.586	0.888	0.873	0.687
Scale 2	0.817	0.787	0.595	0.893	0.874	0.690
Scale 3	0.830	0.800	0.600	0.900	0.883	0.700
All	0.831	0.801	0.607	0.901	0.885	0.703

**Table 7:** Comparison of *MSDF-IQA* to the state-of-the-art on authentic distortions (CLIVE [34] and KonIQ-10k [1]). Median PLCC, SROCC, and KROCC values were measured over 100 random train–test splits. Best results are typed in bold, and second best results are typed in italic.

	CLIVE [34]			KonIQ-10k [1]		
Method	PLCC	SROCC	KROCC	PLCC	SROCC	KROCC
BLIINDER [23]	0.782	*0.763*	0.576	*0.876*	0.864	*0.668*
DeepRN [40]	*0.784*	0.753	*0.579*	0.866	*0.880*	0.666
BLIINDS-II [11]	0.473	0.442	0.291	0.574	0.575	0.414
BMPRI [41]	0.541	0.487	0.333	0.637	0.619	0.421
BRISQUE [3]	0.524	0.497	0.345	0.707	0.677	0.494
CurveletQA [42]	0.636	0.621	0.421	0.730	0.718	0.495
DIIVINE [43]	0.617	0.580	0.405	0.709	0.693	0.471
ENIQA [44]	0.596	0.564	0.376	0.761	0.745	0.544
GRAD-LOG-CP [45]	0.607	0.604	0.383	0.705	0.696	0.501
NBIQA [46]	0.629	0.604	0.427	0.771	0.749	0.515
PIQE [47]	0.172	0.108	0.081	0.208	0.246	0.172
OG-IQA [48]	0.545	0.505	0.364	0.652	0.635	0.447
SSEQ [49]	0.487	0.436	0.309	0.589	0.572	0.423
*MSDF-IQA*	**0.831**	**0.801**	**0.607**	**0.901**	**0.885**	**0.703**

**Table 8:** Comparison of *MSDF-IQA* to the state-of-the-art on authentic distortions (SPAQ [2]). Median PLCC, SROCC, and KROCC values were measured over 100 random train–test splits. Best results are typed in bold, and second best results are typed in italic.

	SPAQ [2]		
Method	PLCC	SROCC	KROCC
BLIINDER [23]	*0.872*	*0.869*	*0.683*
DeepRN [40]	0.870	0.850	0.676
BLIINDS-II [11]	0.676	0.675	0.486
BMPRI [41]	0.739	0.734	0.506
BRISQUE [3]	0.726	0.720	0.518
CurveletQA [42]	0.793	0.774	0.503
DIIVINE [43]	0.774	0.756	0.514
ENIQA [44]	0.813	0.804	0.603
GRAD-LOG-CP [45]	0.786	0.782	0.572
NBIQA [46]	0.802	0.793	0.539
PIQE [47]	0.211	0.156	0.091
OG-IQA [48]	0.726	0.724	0.594
SSEQ [49]	0.745	0.742	0.549
*MSDF-IQA*	**0.900**	**0.894**	**0.692**

**Table 9:** Comparison of *MSDF-IQA* to the state-of-the-art on artificial distortions (TID2013 [39]). Median PLCC, SROCC, and KROCC values were measured over 100 random train–test splits. Best results are typed in bold, and second best results are typed in italic.

	TID2013 [39]		
Method	PLCC	SROCC	KROCC
BLIINDER [23]	**0.834**	**0.816**	**0.720**
DeepRN [40]	*0.745*	*0.636*	*0.560*
BLIINDS-II [11]	0.558	0.513	0.339
BMPRI [41]	0.701	0.588	0.427
BRISQUE [3]	0.478	0.427	0.278
CurveletQA [42]	0.553	0.505	0.359
DIIVINE [43]	0.692	0.599	0.431
ENIQA [44]	0.604	0.555	0.397
GRAD-LOG-CP [45]	0.671	0.627	0.470
NBIQA [46]	0.723	0.628	0.427
PIQE [47]	0.464	0.365	0.257
OG-IQA [48]	0.564	0.452	0.321
SSEQ [49]	0.618	0.520	0.375
*MSDF-IQA*	0.727	0.448	0.311

**Table 10:** One-sided t-test. Symbol ’1’ means that the proposed *MSDF-IQA* method is statistically better than the NR-IQA method in the row on the IQA benchmark database in the column, while symbol ’0’ means that the proposed *MSDF-IQA* performs significantly worse. Symbol ’-’ is used when there is no significant difference.

	CLIVE [34]	KonIQ-10k [1]	SPAQ [2]	TID2013 [39]
BLIINDER [23]	1	1	1	0
DeepRN [40]	1	1	1	0
BLIINDS-II [11]	1	1	1	1
BMPRI [41]	1	1	1	1
BRISQUE [3]	1	1	1	1
CurveletQA [42]	1	1	1	1
DIIVINE [43]	1	1	1	1
ENIQA [44]	1	1	1	1
GRAD-LOG-CP [45]	1	1	1	1
NBIQA [46]	1	1	1	1
PIQE [47]	1	1	1	1
OG-IQA [48]	1	1	1	1
SSEQ [49]	1	1	1	1

**Table 11:** Cross database test. Methods were trained on KonIQ-10k [1] and tested on CLIVE [34]. The best results are typed in **bold**, and the second best ones typed in *italic*.

Method	PLCC	SROCC	KROCC
BLIINDER [23]	*0.748*	*0.730*	*0.503*
DeepRN [40]	0.746	0.725	0.481
BLIINDS-II [11]	0.107	0.090	0.063
BMPRI [41]	0.453	0.389	0.298
BRISQUE [3]	0.509	0.460	0.310
CurveletQA [42]	0.496	0.505	0.347
DIIVINE [43]	0.479	0.434	0.299
ENIQA [44]	0.428	0.386	0.272
GRAD-LOG-CP [45]	0.427	0.384	0.261
NBIQA [46]	0.503	0.509	0.284
OG-IQA [48]	0.442	0.427	0.289
SSEQ [49]	0.270	0.256	0.170
*MSDF-IQA*	**0.764**	**0.749**	**0.552**

**Table 12:** The best results are typed in **bold**, and the second best ones typed in *italic*.

	CLIVE [34]	KonIQ-10k [1]	SPAQ [2]	TID2013 [39]
BLIINDER [23]	1.85	4.67	16.74	1.58
DeepRN [40]	1.31	1.74	5.67	1.30
BLIINDS-II [11]	15.23	47.25	1365.82	11.96
BMPRI [41]	0.29	0.78	21.54	0.24
BRISQUE [3]	**0.03**	*0.11*	3.36	*0.03*
CurveletQA [42]	0.65	1.75	26.65	0.49
DIIVINE [43]	6.99	18.79	543.68	5.27
ENIQA [44]	4.19	13.00	363.22	3.25
GRAD-LOG-CP [45]	**0.03**	**0.10**	**3.05**	*0.03*
NBIQA [46]	6.35	20.07	580.72	5.04
PIQE [47]	*0.06*	0.17	4.58	0.05
OG-IQA [48]	**0.03**	**0.10**	*3.15*	**0.02**
SSEQ [49]	0.41	1.28	36.44	0.33
*MSDF-IQA*	1.45	1.94	5.85	1.34
